# The right to smoke and the right to smoke-free surroundings: international comparison of smoke-free psychiatric clinic implementation experiences

**DOI:** 10.1192/bjo.2021.35

**Published:** 2021-04-16

**Authors:** Tove Freiburghaus, Rie Raffing, Montse Ballbè, Antoni Gual, Hanne Tönnesen

**Affiliations:** WHO Collaborating Centre for Implementation of Evidence-based Clinical Health Promotion, Clinical Health Promotion Centre, Department of Health Sciences, Lund University, Sweden; WHO Collaborating Centre for Evidence-based Health Promotion in Hospitals & Health Services, Clinical Health Promotion Centre, Parker Institute, Bispebjerg and Frederiksberg Hospital, part of the Copenhagen University Hospital, Denmark; WHO Collaborating Centre for Tobacco Control, Cancer Prevention & Control Programme, Catalan Institute of Oncology, Spain; Tobacco Control Research Unit, Bellvitge Institute for Biomedical Research, Spain; CIBER of Respiratory Diseases, Spain; and Addiction Unit, Department of Psychiatry, Neurosciences Institute, Hospital Clínic de Barcelona, Spain; Addiction Unit, Department of Psychiatry, Neurosciences Institute, Hospital Clínic de Barcelona, Spain; WHO Collaborating Centre for Implementation of Evidence-based Clinical Health Promotion, Clinical Health Promotion Centre, Department of Health Sciences, Lund University, Sweden; and WHO Collaborating Centre for Evidence-based Health Promotion in Hospitals & Health Services, Clinical Health Promotion Centre, Parker Institute, Copenhagen University Hospital at Bispebjerg and Frederiksberg, Denmark

**Keywords:** Qualitative research, carers, comorbidity, human rights, in-patient treatment

## Abstract

**Background:**

In Scandinavia, people with a severe mental disorder have a reduced life expectancy of 15–20 years compared with the general public. Smoking is a major contributor, and smoke-free policies are increasingly adopted in psychiatric clinics around the world. We compared potential facilitators and barriers among staff and management, for the implementation of smoke-free psychiatric clinics.

**Aims:**

To investigate the attitudes and experiences regarding smoke-free policies among managers and staff involved in the implementation processes of smoke-free psychiatric clinics at hospitals in Malmö (Sweden) and Barcelona (Spain).

**Method:**

We used a qualitative methodology, with 15 semi-structured interviews. The interviews were conducted with each participant individually, and were subsequently transcribed. The data were analysed with systematic text condensation.

**Results:**

There were notable differences in how the smoke-free policies were carried out and experienced, and attitudes regarding the policy changes differed in the two settings. Key differences were the views on the right to smoke in compulsory care and to stay in smoke-free surroundings supported by smoking cessation intervention; the prioritisation of staff facilitation of smoking breaks; and views on smoking and smoke-free psychiatry. In contrast, participants agreed on the importance of staff education and management support. A smoking ban by law and belonging to a network of smoke-free hospitals were also relevant.

**Conclusions:**

Staff education, and support from staff and management for the patients’ right to stay in smoke-free surroundings, facilitated successful implementation of smoke-free policies in the psychiatric clinics, whereas supporting the right to smoke was a barrier.

People admitted to psychiatric departments have a two- to three-fold increased mortality compared with the average population.^1^ In Nordic countries, severe mental illness reduces life expectancy by 20 years for men and 15 years for women, and smoking is a major contributor.^1^ Other factors affecting mortality in the population with mental illnesses are sedentary lifestyle, obesity, alcohol and drug misuse, socioeconomic marginalisation and inadequate medical care of somatic illnesses.^2–4^ The number of smokers, cigarettes smoked per day and years of smoking are all increased in people with mental illness compared with the general population.^5^

## Smoking and mental care

Professionals in mental care often see tobacco as self-medication.^6^ However, research indicates that smoking negatively affects psychiatric treatment, and smoking cessation supports mental recovery^7^ by having a positive effect on psychological quality of life, with one study indicating that it reduces readmission to psychiatric hospital.^8^ Other studies found no worsening of symptoms of mental illness following smoking cessation for patients with mental disorders, including anxiety disorders, schizophrenia, depression and post-traumatic stress disorder.^9–11^ However, smokers with mental illness have a greater likelihood of experiencing more severe nicotine withdrawal symptoms when quitting compared with the general population; hence, without enough treatment, this can be misinterpreted as a worsened mental health condition.^12^ An effective smoking cessation intervention (SCI) combines pharmacological and intensive behavioural treatments^8,13^ for all patients, including those with severe mental disorders. The potential for health gains resulting from smoking cessation is great, and smoke-free policies and strategies for intervention are increasingly being adopted in psychiatric wards worldwide. The wording smoke-free and not tobacco-free is used intentionally in this context, because the focus is not on other tobacco types common in Sweden, where, for example, 20% of the background population uses snuff. The World Health Organization recommends all healthcare facilities and immediate surroundings to be smoke-free, a policy increasingly adopted globally. However, within mental care, such policies are often faced with resistance, and psychiatric wards may therefore be exempt from legislations on hospital smoking bans or smoke-free policies.^14^ Reasons for this can be varied, but issues have been raised, such as a smoking ban being unethical according to human rights, and being unsafe for staff.^14^ Today, smoke-free policies have been put in place indoor in multiple psychiatric units across the world, although an indoor and outdoor smoking ban is less frequent.

### The Catalonia Region

Spain was one of the first countries to pass laws banning smoking both indoors and outdoors in all healthcare facilities, including all psychiatric wards, in January 2011. The change has been perceived as successful and has become a reality despite initial concerns.^15^ At the Hospital Clínic de Barcelona, a smoke-free policy supported by an SCI with free pharmacological support was implemented in the psychiatric ward in 2010, a year before the national law was implemented (Table 1). The Hospital Clínic de Barcelona is a member of the Catalan Network of Smoke-free Hospitals since 2005, and leads a working group of the Network about Tobacco and Mental Health. The working group comprises 26 professionals from mental health wards of different hospitals in Catalonia, with the aim to improve tobacco control strategies in mental healthcare facilities.

**Table 1:** Timeline on implementing smoke-free policy in psychiatric wards

	Hospital Clínic de Barcelona	Psychiatry Clinic at Skåne University Hospital
Decision to begin process toward a smoke-free psychiatric wards	2010	2012
Smoking ban by law in acute mental health wards (both indoor and outdoor areas included)	2011	No
Programme for tobacco cessation aimed at staff	2005	2012
Staff education: tobacco cessation intervention courses	2010	2013
Programme for tobacco cessation aimed at patients	2006	2015
Closing smoking rooms for patients	2010	2015, Incomplete
Installation of smoking booths inside psychiatric wards	No	2015
Project on smoke-free hospital areas and closing outdoor booths	–	2016

### The Skåne Region

The 2004 Swedish tobacco law allowed smoking in parts of venues intended for healthcare if they were used only for that purpose. An updated law banning smoking in all healthcare facilities was put into effect in July 2019. Nevertheless, smoking was allowed in designated areas. Although not obligated by the law, most healthcare providers support the Swedish Tobacco End Game 2025 initiative, defined as a smoke rate <5% in the population.^16^ In Southern Sweden, the county council Region Skåne and the Malmö Psychiatric Clinic at Skåne University Hospital also support the Tobacco End Game, and originally included psychiatric wards in their early regional policies. In 2012, Malmö Psychiatry initiated a project to implement smoke-free psychiatric wards. This included a local policy that provided free SCI for staff and patients, using the Gold Standard Programme^13^ and education of staff to conduct SCI and implement the tobacco policy. However, in 2015, another policy was implemented allowing smoking booths inside psychiatric wards again, because of staff requests (Table 1).

The implementation literature comparing experiences, facilitators and barriers of smoke-free psychiatric wards across countries is sparse. This study was undertaken to expand this knowledge based on staff and management with the potential power to influence policies and implementation. The aim was to compare potential facilitators and barriers for implementation of smoke-free psychiatry among staff and management in two settings: Malmö, Sweden and Barcelona, Spain.

## Method

The study was performed during 2017, using a qualitative design and semi-structured interviews.^17^ Overall, nine of 13 possible participants in Malmö and six of 14 possible participants in Barcelona were interviewed (Table 2); one pilot interview was conducted. The reasons given for declined participation were no response/no reason, no longer working in the ward, limited knowledge of the topic and language barriers.

**Table 2:** Information about participants

Participant	Position	Gender	City
M1	Management	Woman	Malmö
M2	Staff	Woman	Malmö
M3	Management	Woman	Malmö
M4	Staff	Man	Malmö
M5	Management	Woman	Malmö
M6	Management	Woman	Malmö
M7	Management	Man	Malmö
M8	Staff	Woman	Malmö
M9	Management	Woman	Malmö
B10	Staff	Man	Barcelona
B11	Management	Woman	Barcelona
B12	Management	Man	Barcelona
B13	Management	Man	Barcelona
B14	Management	Woman	Barcelona
B15	Staff	Woman	Barcelona

We contacted members of the management and healthcare staff working with implementation of health promotion in the psychiatric clinic at Skåne University Hospital, Malmö and Hospital Clínic in Barcelona, Spain. Participants were initially identified through the respective public websites.

A preparatory interview was held with the first author to highlight potential preconceptions and bias when performing interviews. All interviews were conducted individually in Swedish or English at local offices. They lasted 20–30 min and were recorded for subsequent transcription. Interview guides were developed together with an anthropologist and aimed to explore general themes related to personal attitude toward a smoke-free psychiatric ward, experiences of implementing a smoke-free policy, facilitators and barriers, organisation and decision-making. During interviews, public organisational diagrams were used to help participants explain how decisions were made and implemented at their hospital and ward.

### Analyses

After transcription, systematic text condensation was used because it is intended for healthcare research and is a suitable way to summarise the experiences of individual informants.^17^ Interviews in Malmö and Barcelona were analysed separately and subsequently compared using the QSR NVivo Plus Qualitative Data Analysis Software for Windows. Data were analysed in six steps. First, we conducted a data overview to identify preliminary themes. We prioritised six themes for further analysis. Second, data were decontextualised, where meaning units were grouped related to the six themes. Third, we condensed the meaning units into subgroups, and further into ‘artificial quotes’. An original quote conveying a similar message as the condensate was then identified. Fourth, we recontextualised text created from the condensates and original quotes. The meaning units of each code group were controlled for being representative and illustrative. Fifth, we compared the analytical texts from Malmö and Barcelona; differences and similarities were identified, forming the categories in the *Results* section. Finally, data were validated by re-reading original transcriptions to look for parts challenging the conclusions.

### Ethics

No patients were included and no material from medical or staff records was used. Participation was voluntary and participants received detailed study information, including information on how the material from the interviews would be anonymised before analyses. Because of staff management being public, complete anonymisation could not be guaranteed for this group. All participants agreed to this and gave informed consent. Therefore, no ethical approval was deemed necessary.

## Results

The six prioritised themes fell into four categories (see A–D in Table 3).

**Table 3:** Summary of results after the analyses based on systematic text condensation (compulsory care: mandated by a judge or voluntarily accepted by the patient)

Malmö	Barcelona
A. Experiences related to smoke-free policies in psychiatry	
Partial smoking ban Staff follow patients in compulsory care to smoke outside Negative experiences: unsuccessful implementation Smoke-free psychiatric wards are not possible Implementation of smoking booths	Total smoking ban Patients in compulsory care are not allowed to smoke Positive experiences: successful implementation Smoke-free psychiatric wards are possible
B. Decision-making and implementation: facilitators and barriers for implementing a smoke-free psychiatric ward	
Participants agreed that attitudes among staff are a barrier Participants agreed that education of staff is important Participants all thought that management support is important Some participants in Malmö and Barcelona thought of uniform implementation as important Participants all thought economy should not be an important factor	
Reorganisation might have affected implementation of smoke-free policy Compulsory care viewed as the main barrier to a smoke-free psychiatric wards Staff take patients outside to smoke Need for staff to take patients outside was seen as costly	Expert units might be important regarding organisation Compulsory care not seen as a barrier Patients are not taken outside to smoke Having the ban be a law was seen as important but not sufficient Being a part of a network of smoke-free hospitals was seen as important Staff taking patients outside to smoke was seen as problematic
C. Participants’ views on smoking and smoke-free psychiatric wards	
Participants agreed that smoking is a problem among psychiatric patients and a cause for premature mortality Participants in Barcelona and some in Malmö thought smoke-free environments were important to aid smoking cessation	
Patients have the right to smoke in compulsory care Focus of in-patient care should be on mental illness Patients need to smoke and do not want to quit Tobacco cessation for out-patients only Smoking bans are illusory	Patients have the right to smoke-free surroundings Discrimination to exclude psychiatric patients from smoke-free policy Psychiatric wards should be completely smoke-free Desire to reduce health inequalities
D. Comparison with policy on other substances	
Alcohol consumption can be regulated by law in Sweden Smoking not seen as a diagnosis	Smoking considered comparable to alcohol Smoking seen as an addiction and illness

### Experiences related to smoke-free policies in psychiatric wards

In Barcelona, participants generally viewed the policy as being accepted among staff, even among those who initially had been reluctant to the change. The same was said about patients: they accepted the policy, incidents were rare, and patients showed no worsening in mental health as a result of smoking cessation.

‘We decided as it is therapeutically work […], it should have the same norms as the rest of the admission. And we forbade to smoke all the time during the admission’ (B11).

All participants agreed with the following:

‘I think that today nobody complains about it. So […] this is the best proof that it's been a very successful approach because now this is accepted as standard care’ (B12).

In Malmö, participants said that, on wards mixing patients with and without compulsory care, the situation became chaotic as the compulsory care patients could not go outside alone.

‘One caretaker told me how many times she unlocked and opened the door to let in those who had been out smoking. I think she said up to 100 times per evening shift’ (M7).

Incidents were also mentioned where patients had become aggressive and were moved to a smoking room available in the psychiatric intensive unit. Some participants said about a smoke-free psychiatry:

‘It is a goal to aim for but to get there is hard, to not say impossible. Unfortunately’ (M4).

Others expressed:

‘It is a balance between working environment for our co-workers who signalled that it is not possible without smoking booths. We cannot make it. […] No, I think we have to accept smoking booths even if we didn't want to’ (M3).

### Decision-making and facilitators and barriers to implementation

Both in Barcelona and Malmö, the participants agreed that staff attitudes were a barrier:

‘I am a big opponent to smoking. But it is hard in the psychiatry to get rid of it. There are so many beliefs among the staff here: that it is hard to quit smoking for our patients and that they will get ill, that there will be more violence’ (M1).

‘Mental health professionals tend to concentrate on what they consider mental health problems. But not other health issues affecting their patients’ (B10).

Additionally, the need for management support was put forward in both places:

‘What is needed is a clear management support for it. […] Then you have to work with the culture. I think within the psychiatry people tend to think that smoking is a comfort and relieves anxiety’ (M8).

‘I think that the pressure from the head of the service was important’ (B12).

Most participants agreed that an important factor was education, but not economy.

In Barcelona, participants agreed that compulsory care was not a barrier:

‘The same as the other patients. Because we thought all of us that for that kind of patient it was impossible to understand the rules and the laws in a place and that they are not able to understand that and to comply with things. But that's not at all! They understand that’ (B15).

There had been initial arguments that it was not right to ban smoking among patients in compulsory care, but now participants clearly opposed this:

‘It's not a right, it's the opposite. They should have the right to stop smoking. The right is to be treated for an illness. The hospital has to be a smoke-free environment. […] it's the right of the smoker to be in a safe place and to able to be helped to stop smoking’ (B13).

The Spanish law banning smoking in all healthcare facilities and belonging to a network of smoke-free hospitals were considered important facilitators.

In Malmö, compulsory care and taking patients outside to smoke were seen as the main barriers. Some participants mentioned that a recent reorganisation had affected the implementation:

‘The reorganisation is still not done everywhere. […] It needs to be done before you can start bigger changes’ (M1).

### Participants’ views on smoking and smoke-free psychiatric wards

At both hospitals, the participants agreed that smoking is bad for the health and causes premature mortality:

‘Our patients live 15–20 years shorter lives and smoking is a big contributor’ (M4).

Additionally, participants agreed that smoke-free environments facilitate implementation. The participants expressed the importance of patients’ rights in general.

In Barcelona, many expressed that psychiatric patients should not be treated differently, but have the same rights to smoke-free surroundings as other patients:

‘This [a smoke-free psychiatric ward] needs to be seen as standard care. That's nothing special, it's something you aim for in all the hospitals around the world. And there is no reason why psychiatric patients should be treated in a different way’ (B12).

In Malmö, most participants talked about the patients’ right to smoke in compulsory care. There were differing views on why this would be considered a right; some referred to the policy decision on the issue that was made in 2015, and a few referred to the increased right, by law, for patients in compulsory care. Some expressed that it would be a violation to ban smoking:

‘I think it is quite a big violation too. First, we take the patient in. You can't do this, you can't do that. How much should we take away from the patient?’ (M7).

Some expressed that focus of care should be on mental illness and not on smoking cessation, whereas several participants mentioned that smoking is calming for the patients and the one thing they live for:

‘I think it is quite controlling, to say “now you are not allowed to smoke. End of conversation.” Many of our patients, that's one of the few things they enjoy doing. I am not saying it is good. And I am not saying that one shouldn't work with smoking and alcohol, but you have to consider the patients, what is best for them’ (M7).

Several participants suggested that work with smoking cessation should be conducted mainly in out-patient settings.

### Comparison with policies on other substances

Some participants in Malmö and Barcelona compared tobacco with other substances, but had differing views on the two issues. In Barcelona, the participants referred to smoking as an addiction disorder and diagnosis:

‘I think it's the concept, which is very clear - it's an illness. You would not allow cocaine, right? Or alcohol. […] It's the same thing. It's not so much about … It's also important that people who don't smoke don't get exposed to smoke. But it's not just that. It's about the patients themselves. They come here to heal. They don't come here to get worse’ (B13).

In Malmö, some referred to the Swedish law on compulsory treatment in healthcare to enable patients to stop drinking alcohol. The matters were considered to differ in that alcohol is an addiction, but smoking could not be diagnosed:

‘If you are a smoker it is not a diagnosis. But if you are addicted to alcohol and impaired there are diagnoses’ (M5).

## Discussion

Today, implementation of smoke-free psychiatric wards is still a challenge in many countries, including Sweden, although successes exist such as Uppsala University Hospital. This study identified several barriers and facilitators for implementation at hospitals in Malmö, Sweden, compared with Barcelona, Spain. Key differences included views on patients’ right to smoke in compulsory care versus right to smoke-free surroundings and receive SCI support. Other differences were prioritising staff facilitation of smoking breaks, views on smoking and smoke-free psychiatry, experiences with patient aggressions, focus on mental care and alignment between policies on smoking and alcohol drinking. The participants agreed on the need for staff education, management support, uniform policy implementation, positive staff attitudes and that economy should not be a limiting factor.

### Attitudes

Few other studies have also noted the importance of not using staff to take patients outside to smoke. One study at a mental health trust concluded that staff facilitation of smoking was a main cause of incidents, and resulted in strained staff resources and patient agitation.^18^ Another study showed that the implementation was compromised by giving permission to smoke and spending time taking patients out smoking.^19^ Furthermore, partial smoking bans that allowed patients to smoke in designated areas have been reported to be less successful than total smoking bans. They undermine clinical management of nicotine dependence, lead to a maintained smoking culture in the wards and cause conflicts between staff members and between patients and staff.^14^ These findings agree with the results of this study.

In Malmö, there were also descriptions of potentially dangerous situations occurring, where patients had become aggressive following the smoking ban. In Barcelona, the experience was the opposite, as incidents were rare. Previous studies have reported that the most perceived barrier among professionals in mental care is the fear of patient aggression and non-adherence.^20^ However, although members of staff often fear that violence and aggression among patients should increase, actual incidents rarely increase; they often stay unchanged after implementation of a smoke-free policy, or may even drop significantly.^21^

### Decision-making and facilitators and barriers to implementation

Another key finding was the supportive view on patients’ right to smoke in compulsory care in Malmö. In contrast, the Northern Ireland Human Rights Commission stated two decades ago: ‘No treaty or other instrument defines a human right to smoke, and the Commission does not accept the position sometimes advanced by the tobacco lobby that there is such a human right’.^22^ In 2008, a high court in England ruled against the case that patients in compulsory care should be provided with opportunities to smoke. Judges rejected the claim that a smoking ban was discriminatory under the European Conventions of Human Rights.^23^

Claiming the right to smoke should be balanced with the right for patients to be in safe and health promoting environments. In line with this, participants in Barcelona stressed that patients in psychiatric care should be given equal rights as somatic patients to a smoke-free hospitalisation.

### Participants’ views on smoking and smoke-free psychiatric wards

Several studies have demonstrated that tobacco-free policies can motivate smokers to quit: a systematic review showed an increased smoking cessation rate of 6.4 percentage points in worksites with tobacco-free policies compared with those without.^24^ Additionally, smoke-free psychiatric wards and hospitals have been shown to increase the motivation to quit, the number of quit attempts and a decrease in the number of cigarettes smoked daily.^25^ This was reflected in Barcelona, where a smoke-free status during stay was seen as one step in an ongoing effort toward quitting entirely.

Some structural differences were considered important for successful implementation in Barcelona. They included a uniform law banning smoking in all healthcare facilities, management support, belonging to a network of smoke-free hospitals and having an organisational structure where the decision makers received input from experts in all parts of the psychiatric ward. Consistency, cohesive leadership and full administrative support have been reported to be essential for the successful implementation,^26^ but further research is needed to establish the importance and interaction of the other structural factors.

### Comparison between tobacco and alcohol

In Barcelona, smoking was described as an addiction and an illness in line with alcohol use disorder, whereas members of management in Malmö said that smoking was not a diagnosis. This is contradictory to the international diagnostic manuals used in psychiatry in Sweden and worldwide.^27^ To regard smoking as a diagnosis would be a crucial step toward seeing smoking as important within mental care. In Malmö, some participants expressed that smokers with mental illness do not want to quit or that they need to smoke. However, people with mental disorders seem to be almost as motivated to quit as the general population,^28^ and only minor differences have been reported in successful quitting among smokers with and without mental illness over 6 and 12 months.^13^

### Bias and limitations

This study has several biases, such as lack of documentation regarding actual incidents related to smoking situations and the smoking status of the participants, which may affect their views. Interview time in Barcelona was shorter, which may have influenced the number of interviewees, and they were performed in English and not the local language, which was in contrast to the interviews in Malmö and may introduce a selection bias in favour of alignment with other English cultures, where widespread implementation of tobacco-free psychiatric hospitals is more common. A strength is that this study investigated two comparable countries, as both Spain and Sweden are high-income countries and the hospitals were actively implementing smoke-free policies. However, this means that the results may be limited to mental care settings like the ones described. There is a general limitation inherent to the interview method because of the generalisation made from qualitative interviews and the effect of the researcher's personal bias.^29^ The latter concern, however, was addressed by pre-interviewing the interviewers and pilot testing the interview guide. The use of transcribed text from the interviews has been criticised for its potential to change the inherent meaning of the contextual reality in which the interviews took place.^30^ Despite this criticism, the method is regarded as a strong research tool to obtain powerful data on experiences if the researchers, as in this project, are careful not to make their own interpretations of the interview data. In addition, the validity of the results was substantiated by the systematic methodology used, including the final validation (stage six), described in the *Method*.^29^

### Clinical implications

These results may aid psychiatric wards and hospitals in the process of implementing a smoke-free policy to benefit the patients and staff. The need for education, management support, uniform implementation and encouragement of positive staff attitudes require dedicated actions in line with other policy implementations. Comparing two countries adds to previous reports by shedding a broader light on the different attitudes, experiences and views on patients’ right to smoke or to stay in smoke-free surroundings, and implementing smoke-free psychiatry. This knowledge affects the culture, attitudes and behaviour as part of the capacity building of a learning organisation. In the long run, it may contribute to the important work on reducing the severe health inequality and premature mortality among people with severe mental illness.

### Implications for future research

Future research should evaluate specific implementation strategies to expand the knowledge in this important area. An interesting comparison could be with the well-implemented policy on alcohol-free psychiatric wards.

In conclusion, this study showed that problems with implementation of smoke-free psychiatric wards may arise because of staff facilitation of smoking breaks, the implementation of a partial smoke-free policy, viewing compulsory care as a barrier, prioritisation of patients’ right to smoke in contrast to their right to be in a smoke-free environment, and failing to view mentally ill patients equally to other patients. Factors of potential importance for the implementation were a total smoke-free policy, management support, education of staff, a smoking ban by law, belonging to a network of smoke-free hospitals and a psychiatric organisation with clinical expert groups.

## Data Availability

The study data consists of transcribed interviews that are not publicly available because potentially disclosing interviewee identifiable information would be unethical.

## Supplementary material

For supplementary material accompanying this paper visit http://dx.doi.org/10.1192/bjo.2021.35. click here to view supplementary material
